# Designed construction of tween 60@2β-CD self-assembly vesicles as drug delivery carrier for cancer chemotherapy

**DOI:** 10.1080/10717544.2018.1440448

**Published:** 2018-02-20

**Authors:** Yue Yuan, Qin Zhang, Yun Yan, Miaomiao Gong, Qi Zhao, Zhihong Bao, Kaerdun Liu, Siling Wang

**Affiliations:** a School of Pharmacy, Shenyang Pharmaceutical University, Shenyang, P. R. China;; b Beijing National Laboratory for Molecular Sciences, College of Chemistry and Molecular Engineering, Peking University, Beijing, P. R. China

**Keywords:** Self-assembly, vesicles, DOX, encapsulation efficiency, cellular uptake

## Abstract

We report a simple strategy to prepare Tween 60@2β-CD self-assembly vesicles in aqueous solution as a new drug delivery carrier for cancer chemotherapy. The spherical shape of vesicles was confirmed by transmission electron microscopy (TEM) and mean particle sizes were about 33.7 nm, as measured by dynamic light scattering, micro-IR results indicated that the self-assembly vesicles was driven by hydrogen bonding. Hydrophilic doxorubicin (DOX) was successfully loaded into the self-assembly vesicles with drug loading content of 7.85% and loading efficiency of 42%. In addition, an *in vitro* cytotoxicity study and cellular uptake assays demonstrated that the DOX-loaded Tween 60@2β-CD vesicles markedly enhanced the cellular uptake and cytotoxicity of DOX toward the Hela cells. Furthermore, when used to evaluate the *in vivo* therapeutic efficacy in mice bearing the breast cell line (4T1), DOX-loaded vesicles exhibited superior inhibition of tumor growth compared with the DOX solutions.

## Introduction

Molecular self-assembly has exhibited as a smart strategy for the preparation of well-defined nanoscale supramolecular structures. Driven by kinds of inter-molecular non-covalent interactions such as hydrogen bonding (Zhong et al., 2016; Gao et al., 2017), host-guest interaction (Tao et al., 2012; Valente & Soderman, 2014), electrostatic force (Jian et al., 2017; Mantha et al., 2017), and aromatic conjugation (Dou et al., 2006; Ma et al., 2010). In contrast with covalent bonding, the non-covalent interactions make self-assemblies different from supramolecular aggregation both in structure and property. The advantages make them very competitive applicants for exploiting smart materials in diverse fields such as controlled delivery (Liu et al., 2011; Chen et al., 2016; Wang et al., 2016), voltage sensors(Bond & Sansom, 2007), or stimuli responsive materials (Oueslati et al., 2014; Ganta & Chand, 2015). One of the most rapidly developing areas of functional materials nowadays is nanoscale vesicle (Yao et al., 2007; Zhang et al., 2013; Li et al., 2015; Geng et al., 2016). Vesicles with hollow space inside and spherical structure are suitable nanocarriers for intracellular drug delivery due to their inherent characteristics, including high stability and tunable amphiphilicity of the building blocks. Although there are many reports on the self-assembly vesicles, it is very difficult to prepare this system with low weight and biocompatible molecules in the vesicles formation process. In addition, there was no scientific evaluation for the drug delivery of self-assembly vesicles. So, it remains a great challenge to utilize drug loaded self-assembly vesicles in cancer chemotherapy, or prepare the system of low weight and biocompatible molecules through a facile method.

In recent decades, synthetic nanomaterials, including inorganic microballoon sphere, polymers, or nanoparticles have been intensively researched as drug-delivery carriers for the treatment of cancers (Prakash Jain et al., 2011; Zhang et al., 2015; Han et al., 2016). Among the drug carriers for cancer chemotherapy, surfactant-based vesicles are the most extensively studied and they possess the most suitable characteristics to be converted into functional devices, with a better chemical stability and a low cost of production. (Tavano & Muzzalupo, 2016; Li et al., 2017). These increase therapeutic index and reduce toxicity by spontaneous drug accumulation at tumor site through the enhanced permeability and retention (EPR) effects. However, the main drawback of these synthetic nano-carriers is their slow degradation within human body. As we all know that cyclodextrins (CDs) are cyclic oligosaccharides composed of α-1,4-coupled-D-glucose units (Jiang et al., 2011b; Yan et al., 2011), and CDs have been widely used in drugs and cosmetics for its safe degradation by α-amylase with remarkable bio-compatibility (Fetzner et al., 2004; Miranda et al., 2011). CDs and most surfactant can form complexes by host-guest interaction, and CDs can transform surfactant micelles into well-defined structure such as vesicles, microtubes, lamellar structure (Jiang et al., 2011a).

In our previous report (Zhou et al., 2013), the self-assembly vesicles formed from Tween 20/β-CD inclusion complex have been prepared, however, the drug-loaded vesicles have not been investigated systematically. In recent study, it was found that the particle sizes of vesicles formed by Tween 60/β-CD were smaller and more uniform than that of Tween 20/β-CD. Moreover, Tween 60 is less toxic to human body as compared with Tween 20 among the non-ionic surfactants. The former had higher entrapment efficiency and could deliver drugs to tumor site by EPR effect. Therefore, we developed a novel flexible, biodegradable, and self-assembly Tween 60/β-CD vesicles that can ultimately serve as controlled drug delivery for tumor targeting in this study.

The most commonly reported approach for the preparation of vesicles was film dispersion method (Guo, 2006; Prabhakara et al., 2013; Suga et al., 2014). However, this method has some limitations such as the complicated synthesis process, larger diameters, and more organic solvents involved, which may limit the vesicle’s application in drug delivery. To address the above challenges, here we report the synthesis and drug-carrier application of surfactant/β-CD vesicles in aqueous solution. Conceptually, Tween 60/β-CD self-assembly vesicles may offer some key advantages in comparison to liposomes. Tween 60/β-CD self-assembly vesicles are expected to be significantly more stable than liposomes, which were composed by phospholipid suffering from oxidation easily. Besides, the preparation process of Tween 60/β-CD self-assembly vesicles are completely in aqueous solution without any organic solvents. Finally, the diameter of the self-assembly vesicles was suitable to passive targeting to tumor tissue through the EPR effects.

## Materials and methods

### Materials

Tween 60 (Polyoxyethylene sorbitan monolaurate) was obtained from China Pharmaceutical Co. (Beijing, China). β-Cyclodextrin with a water content of 14% was offered by Sinopharm Chemical Reagent Co. (Beijing, China). Doxorubicin (DOX) was purchased from Shanghai Pharmaceutical Co. (Shanghai, China). Ultrapure water was redistilled from potassium permanganate throughout this work.

### Preparation of Tween 60/β-CD self-assembly vesicles

Desired amounts of Tween 60, β-CD and water were added into tubes, and then the samples were vortex mixed sufficiently and heated to obtain isotropic solutions. Then the resultant solutions were thermostatically incubated at 25 °C (for at least 24 h) to allow Tween 60/β-CD system self-assemble sufficiently.

### Physicochemical characterization

#### Transmission electron microscope (TEM)

The morphology of the self-assemblies was observed in a JEOL-100 CX II transmission electron microscope. The samples were prepared with negative staining: a drop of sample was placed on 230-mesh copper grids coated with Formvar film. Excess water was removed with filter paper, followed by negatively staining to film with uranyl acetate. After removal of the excess staining liquid by filter paper, the samples were placed at room temperature to dry for TEM observation. Freeze-fracture transmission electron microscopy (FF-TEM), a small amount of sample was placed on a 0.1 mm thick copper disk and then covered with a second copper disk. The sample was frozen by liquid nitrogen. Fracturing and replication were performed on a freeze-fracture apparatus (BalzersBAF400, Wiesbaden, Germany) at −140 °C. Pt/C was deposited at an angle of 45° to shadow the replicas, and C was deposited at an angle of 90° to consolidate the replicas. The resulting replicas were observed in a JEM-100CX electron microscope. AFM measurements in tapping mode under ambient conditions were conducted on a D3100 AFM (VEECO, Santa Barbara, CA, USA). One drop of the Tween 60/β-CD solution was spin-coated on a mica surface, and then placed at room temperature to dry before AFM observation. FT-IR measurement was performed on a Nicolet Magna IR 750 equipped with an infrared micro-spectrograph (Thermo Scientific Co., Waltham, MA, USA). The vesicle samples were frozen in liquid nitrogen and subsequently lyophilized for 48 h before FT-IR measurements. For 1 H-NMR measurements, the samples were prepared in D2O. All of the measurements were performed on an AVANCE III 500 M Hz NMR (Bruker, Fällanden, Switzerland).

### The drug loading process and determination of the entrapment efficiency

DOX loaded vesicles were prepared by ammonium sulfate gradients method, DOX (1.2 mg mL^−1^) was dissolved in 5 mL ammonium sulfate gradient Tween 60/β-CD vesicles (*C*_Tween 60_ 1 mM, *C*_β-CD_ 2 mM), the system was thermostatically incubated at 40 °C for 30 min to allow DOX loading sufficiently. The unloading DOX was removed by dialysis method. The amount of original and supernatant DOX was analyzed by UV-vis at a wavelength of 480 nm. The DOX entrapment efficiency (EE) and the drug loading content (DLC) were calculated using the following formula (1) and (2), respectively.

(1) $$\text{EE }\left(\%\right)=\left({\text{m}}_{\text{t}}-{\text{m}}_{\text{u}}\right)/{\text{m}}_{\text{t}}\times \text{1}00\%$$

(2) $$\text{DLC }\left(\%\right)=\left({\text{m}}_{\text{t}}-{\text{m}}_{\text{u}}\right)/{\text{m}}_{\text{vesicles}}\times \text{1}00\%$$

Where m_t_ is the total weight of DOX added, m_u_ is the unloaded weight of DOX, and m_vesicles_ is the total weight of vesicles.

### Release behavior of DOX from Tween 60/β-CD self-assembly vesicles

The release study of DOX loaded vesicles was performed in pH 7.4 and pH 5.8 PBS. Formulations (containing DOX relevant to 1.2 mg) were transferred into a dialysis bag (MWCO 3000) and sealed with a dialysis bag holder, the dialysis bags were then immersed into PBS (25 mL) with constant shaking at 100 rpm/min at 37 °C at predetermined time intervals, the release medium (1 mL) was withdrawn to monitor the release extent of DOX by an UV-Vis spectrophotometer (UV-2000, UNICO, Fairfield, NJ, USA) at a wavelength of 480 nm using a pre-established calibration curve. Simultaneously, the fresh medium with the same volume was replenished. The results were averaged with three measurements.

### Cell viability assessment

To study the cytotoxicity of self-assembly Tween 60@2β-CD vesicles, the cells (including hRPE cells and Hela cells) were grown in the presence of the blank vesicles. Cells were seeded into (96-well) plates at a density of 1.0 × 10^4^ cells/well and attached for 12 h. The old medium was replaced with the medium containing different doses of blank vesicles. Untreated cells in growth media were used as the blank control. After 12 h incubation, the cell viability was evaluated by CCK-8 assay.

The *in vitro* cell viability of Hela cells, which were incubated with the DOX-Sol and DOX-encapsulated samples was evaluated by CCK-8 assay. Briefly, cells were seeded into (96-well) plates at a density of 1.0 × 10^4^ cells/well and attached for 12 h. Then the culture medium was removed and cells were subject to DOX-Sol and DOX-Vesicles solutions in serum-free medium at equivalent DOX concentrations of 0.0001, 0.001, 0.01, 0.1, and 0.5 μg mL^−1^ were used to investigate the cytotoxic effect of different DOX formulations. At the time point of 12 h, CCK-8 (10 μL) was added to each well. After being incubated for 2 h, the 96-well plates were finally placed into an iMark™ microplate reader (Bio-RAD, CA, USA) to monitor the absorbance at wavelength of 450 nm. Cells without treatment were set as control. The cell inhibitory rate was calculated using the following formula (3):

(3) Cell inhibitory rate (%) = Abs(test cells)- Abs (reference cells)/Abs(reference cells)×100%

### *In vitro* intracellular uptake

To compare the cellular uptake efficiency of DOX-Sol and DOX-Vesicles, Hela cells were seeded on round glass coverslips in 24-well plates. After 12 h of attachment, the culture medium was replaced by various DOX formulations (containing DOX of 0.01 μg mL^−1^). After incubation for 4 h, the cells were rinsed with cold PBS for three times, and fixed with formaldehyde (4%) for 15 min. The nuclei were afterward stained by Hoechst 33258 for 15 min and the fixed cells were observed using CLSM.

### *In vitro* hemolysis assay

The RBCs were obtained by centrifugation to remove serum and carefully washed with physiological saline until supernatant fluid was clear. Subsequently, 0.6 mL diluted RBC suspension (10%) was added into 2.4 mL Tween60/β-CD self-assembly vesicles to make a series of Tween 60/β-CD vesicles concentrations (10, 50, 100, 250, 300 μg mL^−1^). The mixtures were vortexed and incubated at 37 °C for 2 h. After that, the mixtures were centrifuged at 3000 rpm for 5 min, and the absorbance values of supernatant were measured by a UV-vis spectrophotometer at the wavelength of 570 nm. At the same time, the normal saline and DI water were used as positive and negative control, respectively. The hemolysis percentage was calculated as follows formula:

(4) $$\text{Hemolysis }\left(\%\right)=({\text{Abs}}_{\text{sample}}–{\text{ Abs}}_{\text{negative}})/({\text{Abs}}_{\text{positive}}–{\text{ Abs}}_{\text{negative}})$$

Where Abs_sample_, Abs_positive,_ and Abs_negative_ represent the absorbencies of samples, positive and negative control, respectively.

### *In vivo* antitumor efficacy

All the animal experiments were performed in accordance with the guidelines of the institutional animal use committee. The *in vivo* anticancer efficacy of DOX-loaded vesicles was assessed using a model involving mice with 4T1/KM. When the tumor reached 100–150 mm^3^, the mice were randomly assigned to four groups (*n* = 6), which were given an intraperitoneal injection of physiological saline, blank vesicles, free DOX, and DOX-loaded vesicles (at a dose of DOX 5 mg per kg body weight), respectively, once a day for five days. The tumor volumes and body weights were recorded every other day after treatment and the tumor volume was calculated according to the formula (5):

(5) $$\text{Volume }=\text{a}\times {\text{b}}^{\text{2}}/\text{2}$$

Where a and b are the length and width of the tumor as measured by calipers. After treatment for 10 days, all the mice were sacrificed and their tumors were excised and weighed, and the tumor growth inhibition rate was calculated according to the formula (6):

(6) Inhibition (%)=(C-T)/C×100%

Where C is the average tumor weight of the control group, and T is the average tumor weight of each of the treated groups.

### Statistical analysis

Origin 8.5 (OriginLab Inc., Northampton, MA, USA) was used for the statistical analysis. All experiments were performed at least three times and the data are presented as mean ± SD. Data were analyzed statistically using the Student’s independent sample *t*-test and expressed as one-way *p* value. Significance was accepted for *p*-value of <.05.

## Results and discussion

### Preparation and characterization of self-assembly vesicles

**Figure 1.**
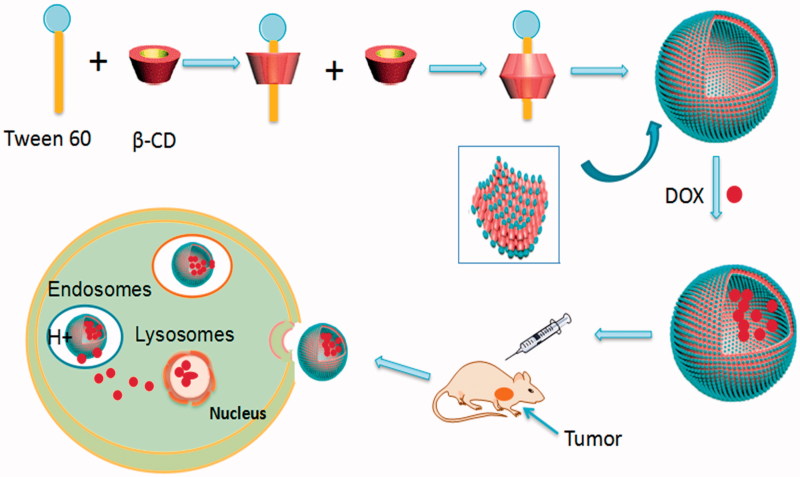
(A) Schematic description of the synthesis of Tween 60@2β-CD self-assembly vesicles loaded with DOX and their accumulation in tumor.

The preparation process of Tween 60/β-CD self-assembly vesicles are shown in Figure 1. The first step is the preparation process of Tween 60/β-CD inclusions. Tween 60 can shelter hydrophobic parts into β-CD cavity by hydrophobic interaction and Van der Waals interactions. The resultant complexes are completely hydrophilic. Then, the Tween 60/β-CD inclusions as building blocks are spontaneously self-assembled into vesicles driven by hydrogen bonding. The whole preparation process was conducted in aqueous solution. The solution of β-CD is transparent at the concentration of 4 mM, whereas the solution is opalescent upon the addition of 2 mM Tween 60 (see the supporting information, Figure S1), indicating the formation of inclusion complexes between Tween 60 and β-CD. The particle size of Tween 60/β-CD self-assembly vesicles was 33.7 nm with the polydispersity index (PdI) of 0.251, which is larger than that of free Tween 60 or free β-CD (Figure S2).

The 1 H-NMR spectra were also widely applied to verify the formation of Tween 60/β-CD inclusion complexes. β-CD molecules are presented as toroid where the H-3 and H-5 protons are located inside the cavity, while the H-2 and H-4 protons are located outside the cavity (Valente & Soderman, 2014; Li et al., 2015). We have known that β-CD cavity is usually occupied by water molecules with absence of proper guest molecules. When adding guest molecules, water molecules were released from the cavity and replaced by more nonpolar guest molecules with suitable sizes. As shown in Figure S3, the chemical shift of H-3 and H-5 protons significantly turned to high-magnetic field, indicating the formation of complex between β-CD and Tween 60.

The binding stoichiometry between surfactant and CDs was determined by the surfactant hydrophobic chain length and the molar radio between surfactant and CDs. Generally, the inclusions at the molar ratio of 1:1 can be formed readily in most cases. But when the concentration of CDs is large enough and the surfactant hydrophobic chain length is longer than 12 C, the 1:2 conclusions form possibly (Zhou et al., 2016). The hydrophobic chain length of Tween 60 is 18 C. Then, further investigation of the ratio between Tween 60 and β-CD is needed. Electrospray ionization-mass spectroscopy (ESI-MS) was utilized to confirm the formation and composition of an inclusion complex (Zhou et al., 2013). The ESI-MS spectrum peak of Tween 60/β-CD [Tween 60@2β-CD +3 H_2_O + 3 K^+^] (*m/z* = 1261.9) and [Tween 60@2β-CD +2 H_2_O + 3 H^+^] (*m/z* = 1217.9) are observed as Fig. S4. The result confirmed the complex of Tween 60/β-CD was formed at the stoichiometric ratio of 1:2.

Next, the IR was conducted to explore the binding sites between Tween 60 and β-CD (Figure S5). The intensity of the 1736 cm^−1^ peak (which is attributed to the stretching vibration of ester carbonyl in Tween 60) was significantly decreased. The peaks at 2921 cm^−1^ and 2853 cm^−1^ are indicative of C-H stretching vibration, and the intensity of those peaks was also clearly decreased. So, the results of IR showed that β-CD was combined at the position of carbonyl and carbon chain in Tween 60.

**Figure 2.**
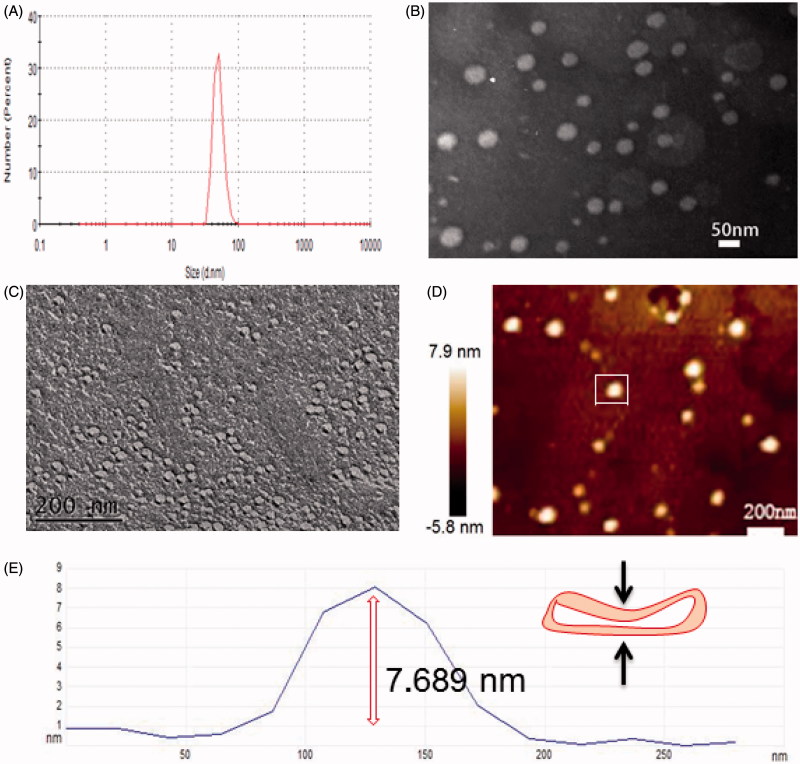
A, B, C, D was DLS, TEM, FF-TEM, AFM image of Tween 60@2β-CD self-assembly vesicles, respectively. E was sectional height profile of a collapsed vesicle chosen in D.

**Figure 3.**
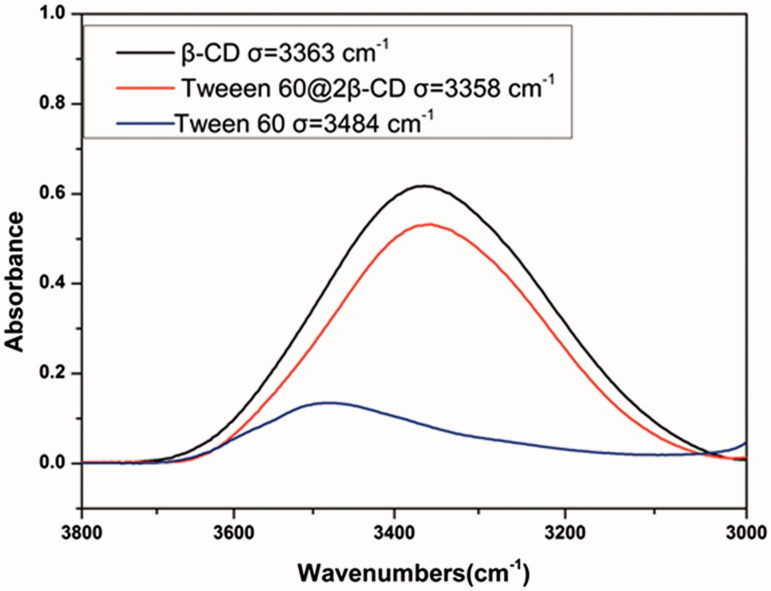
Micro-IR image of Tween 60/β-CD vesicles, Tween 60, and β-CD.

Transmission electron microscope (TEM) and dynamic light scattering (DLS) images (Figure 2(A,B)) showed that Tween 60@2β-CD self-assembly vesicles had a uniform spherical morphology with the average diameter of 33.7 nm. Moreover, the Freeze Fracture TEM (FF-TEM) image (Figure 2(C)) proved that the spherical structure was hollow vesicles rather than solid spheres. Further investigation of the vesicles was carried out with Atomic force microscopy (AFM) (Figure 2(D)). Considering that the height of β-CD is 0.79 nm (Jiang et al., 2011a,b; Zhou et al., 2014a,b) and the extending hydrophobic chain length of Tween 60 is 1.915 nm (Figure S6), the thickness of per Tween 60@2β-CD unit is consistent with the length of Tween 60. The height of vesicle is 7.689 nm (Figure 2(E)), which is about four Tween 60@2β-CD units. That is because the structure can be considered as a collapsed single ventricular vesicle. To investigate the role of hydrogen interaction in the formation of Tween 60@2β-CD self-assembly vesicles, micro-IR was conducted, the results showed that the hydroxyl peak of Tween 60@2β-CD inclusion complex appears at the lower wavenumber of 3358 cm^−1^, whereas the hydroxyl peak of Tween 60 occurs at 3484 cm^−1^ and the hydroxyl peak of β-CD appears at 3363 cm^−1^ (Figure 3). This demonstrated strong hydrogen bonding between Tween 60 and β-CD in Tween 60@2β-CD self-assembly vesicles.

### Drug loading capacity and *in vitro* release

**Figure 4.**
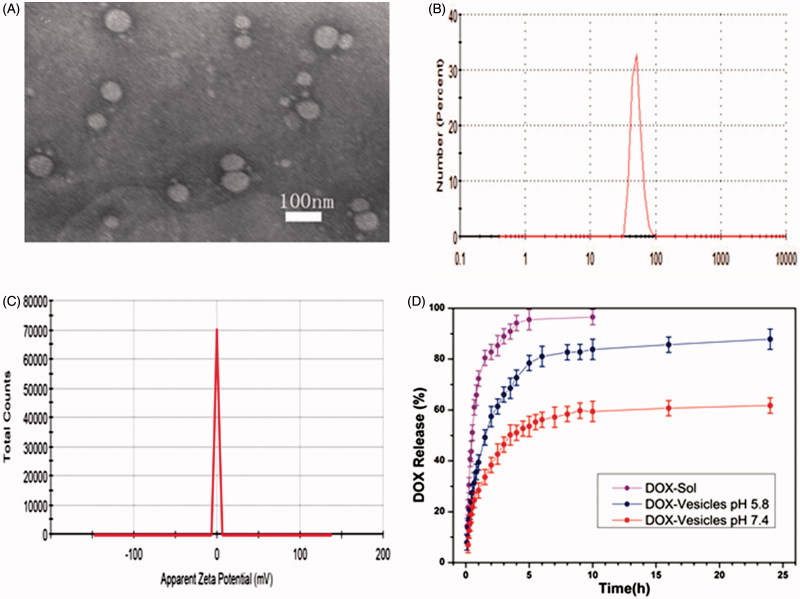
(A) TEM image of DOX-loaded vesicles. (B) DLS image of DOX loaded vesicles. (C) Zeta Potential image of DOX loaded vesicles. (D) *In vitro*, DOX release from vesicles in pH 7.4 and pH 5.8.

Doxorubicin (DOX), one of the first-line chemotherapy drugs for cancer was selected as a model drug. Many approaches toward loading DOX into the well-defined carriers have been developed by making use of the pH-dependent solubility of DOX (Song et al., 2012). We utilized conventional ammonium phosphate gradient method to load DOX into vesicles (Fritze et al., 2006), which has a high-loading efficiency of 42% and the drug loading content of 7.85%. The results of TEM and DLS suggested that the DOX-loaded vesicles (DOX-Vesicles) have an average size of 50 nm with the PdI of 0.194, which is a bit larger than blank vesicles (Figure 4(A,B)). The DOX-loaded vesicles had the zeta potential value of −0.3 mV, which showed that the DOX-loaded vesicles was almost uncharged (Figure 4(C)).

The release behavior of DOX-Vesicles was subsequently investigated *in vitro*. The DOX-Vesicles were placed in pH 5.8 and pH 7.4 PBS and the release profiles are shown in Figure 4(D). For comparison, the release of free DOX solutions (DOX-Sol) was also assessed. DOX-Vesicles exhibited a gradual release of DOX in pH 7.4 PBS and the release percentage was as high as 60% after 24 h. When exposing the DOX-Vesicles to pH 5.8 PBS, the release rate of DOX markedly accelerated and the release percentage at 24 h was 83%. This acid triggered release can be explained as that the electrostatic interactions between the vesicles and DOX were pH-dependent, and DOX molecules tended to detach from vesicles more easily under low-pH conditions. Since the pH value in cancer cells and the endosomal compartment is lower than it in healthy cells (Damaghi et al., 2013), a preferential release of DOX at the tumor site can be achieved. To study the kinetics mechanism of DOX release from vesicles, equations of zero order, first order, Higuchi and Riger–Peppas model were utilized to fit the accumulative release data. The obtained results were listed in Table S1. The highest coefficient (*R*^2^) was observed when the release profiles were described using the Riger–Peppas model, according to the equation, it is feasible to believe that the release mechanism of DOX from these vesicles was the combination of Fick’s diffusion and the disintegration of the skeleton.

### *In vitro* intracellular uptake

**Figure 5.**
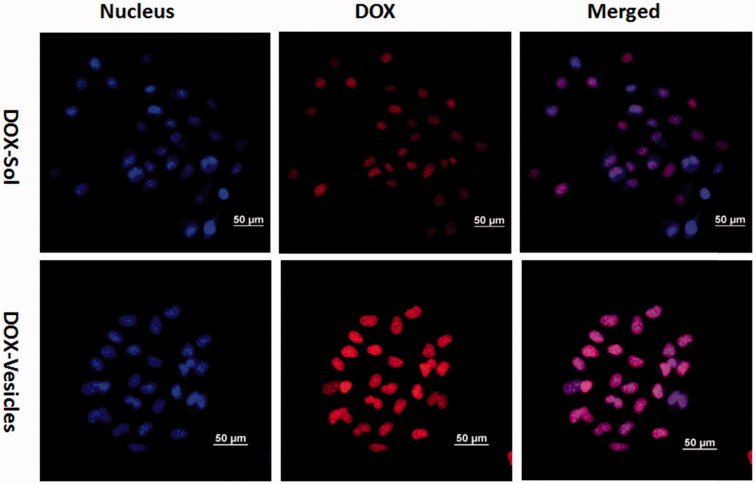
Internalization and accumulation of DOX in Hela cells were conducted with DOX-Sol or DOX-vesicles for 4 h, measured by confocal microscopy. Scale bar is 50 µm.

To investigate the cellular uptake efficiency of the self-assembled vesicles, the DOX-Sol and DOX-Vesicles were incubated with Hela cells for 4 h and the fluorescence signals within cells observed by CLSM were shown in Figure 5. The cell nucleus was counterstained with Hoechst 33258 showing blue fluorescence. Images demonstrated that the DOX fluorescence of DOX-Vesicles was concentrated in cell nucleus regions and the DOX fluorescent intensity of DOX-Vesicles were much higher than DOX-Sol, demonstrating the higher cellular uptake efficiency of DOX-Vesicles for Hela cells. This may be due to uncharged vesicles that could easily approach to the cell surface and triggered an inherent affinity with the cell membrane since they have the similar structure.

### Hemolysis assay

In order to evaluate the security of Tween 60@2β-CD self-assembly vesicles *in vivo* application, hemolysis assay was carried out using rabbit red blood cells (RBCs) from rabbit blood, which is obtained from Laboratory Animal Center of Shenyang Pharmaceutical University. The normal saline and DI water were used as positive and negative control respectively. Compared with DI water, Tween 60@2β-CD self-assembly vesicles showed negligible hemolysis percentage to RBCs at various concentrations range from 10 to 300 μg mL^−1^ (Figure S7). The result revealed that Tween 60@2β-CD self-assembly vesicles have remarkable bio-compatibility and may be a promising drug carrier for vein injection.

### *In vitro* cytotoxicity

**Figure 6.**
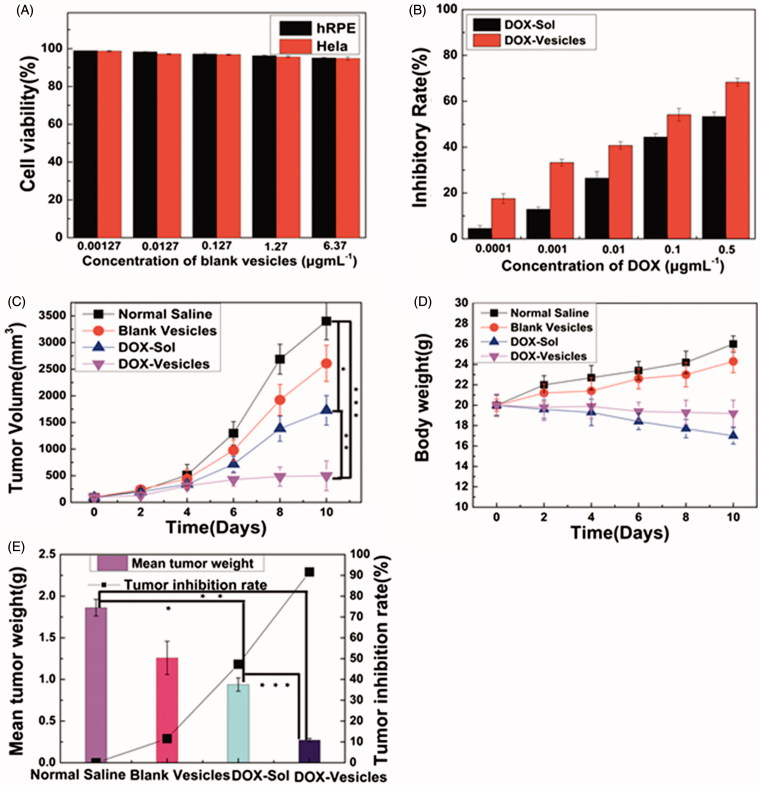
(A) The cell viability of blank vesicles against hRPE cells and Hela cells at different concentrations of Tween 60@2β-CD. (B) Inhibitory rate of DOX-Sol and DOX-Vesicles against Hela cells at different concentrations of DOX. (C) Changes in tumor volume after intravenous injection of normal saline, blank vesicles, DOX-Sol, and DOX-Vesicles. (D) The body weight variations of every group. (E) The tumor inhibition rate and mean tumor weight between groups after 10 days treatment with normal saline, blank vesicles, DOX-Sol, and DOX-Vesicles. (The tumor volume and mean tumor weight between groups was compared by Student’s *t*-test ^★^*p* < .05, ^★★^*p* < .01, ^★★★^*p* < .001).

For drug vesicles, safety is very important for their applications. The cytotoxicity of self-assembly Tween 60@2β-CD vesicles was investigated by using CCK-8, as shown in Figure 6(A). The cell viability of blank vesicles tested on both hRPE cells and Hela cells was usually higher than 90%, which confirmed the good biocompatibility of Tween 60@2β-CD self-assembly vesicles. The profile in Figure 6(B) showed the half maximal inhibitory concentration (IC_50_) of DOX-Vesicles was 0.05 μg mL^−1^ by calculation, while the IC_50_ of DOX-Sol was about 0.2 μg mL^−1^. So, the IC_50_ of DOX-Vesicles was about four-fold lower than that of DOX-Sol. The results showed that the cytotoxic effect of DOX-Vesicles on Hela cells was much higher compared with that of DOX-Sol.

### *In vivo* antitumor efficacy

To investigate the potential of DOX-loaded vesicles inhibiting tumor growth, an *in vivo* therapeutic efficacy study was carried out using KM mice with 4T_1_ liver cancer. The body weight and tumor volume were measured over the whole treatment period of 10 days. As shown in Figure 6(C), the average tumor volume in the groups of normal saline and blank vesicles increased rapidly. Compared with the normal saline, the increase of tumor volume in the animals treated with DOX-Sol and DOX-Vesicles was significantly reduced. Moreover, the group of DOX-Vesicles exhibited higher anti-tumor activity than DOX-Sol, which was a result from the enhanced cellular uptake of DOX-Vesicles by EPR effect and pH-sensitive release at tumor sites. Besides, as shown in Figure 6(D), the body weight of the group treated with DOX-Sol was significantly reduced while the group treated with DOX-Vesicles showed a smaller weight loss, indicating that the incorporation of DOX into the self-assembly vesicles significantly reduced the toxicity of DOX-Sol. After 10 days treatment, the mice tumors were excised and weighed. As shown in Figure 6(E) and Figure S8, the tumor growth inhibition rate of DOX-Sol and DOX-Vesicles was 47.3% and 91.6%, respectively, which was consistent with the tumor volume results. So DOX-Vesicles revealed a better effect of tumor growth inhibition than DOX-Sol.

## Conclusion

In summary, this paper reported a facile approach for directly constructing self-assembly vesicles with surfactant and β-CD in aqueous solution. We found that Tween 60/β-CD complexes can self-assemble into vesicles and the unit of Tween 60@2β-CD was verified to be the building block in well-defined structures. The driving force for the formation of the vesicles is hydrogen bonding. Furthermore, the self-assembly vesicles could encapsulate DOX with a high EE% and release preferentially in the acid environment, the self-assembly vesicles could increase the cytotoxic effect of DOX toward cancerous cells by enhancing the cellar accumulation of the drug loaded vesicles. More importantly, compared with DOX-Sol, DOX loaded Tween 60@2β-CD self-assembly vesicles exhibited excellent tumor growth inhibition with lower side effects. All these results reveal that Tween 60@2β-CD self-assembly vesicle is a promising drug nanocarrier for cancer chemotherapy.

## Disclosure statement

The authors report no conflicts of interest. The authors alone are responsible for the content and writing of this article.

## Supplementary Material

IDRD_Wang_et_al_Supplemental_Content.doc
