# Supplementary material for: Designed construction of tween 60@2β-CD self-assembly vesicles as drug delivery carrier for cancer chemotherapy
Source: Drug Deliv. 2018 Feb 20;25(1):623–31. doi: 10.1080/10717544.2018.1440448 (PMC7025689; doi:10.1080/10717544.2018.1440448)
Supplement: IDRD_Wang_et_al_Supplemental_Content.doc [file IDRD_A_1440448_SM1927.doc]

**Supplementary Information**

**Designed construction of Tween 60@2β-CD self-assembly vesicles as drug delivery carrier for cancer chemotherapy**

Yue Yuan^a^, Qin Zhang^a^, Yun Yan^b^, Miaomiao Gong^a^, Qi Zhao^a^, Zhihong Bao^a^, Kaerdun Liu^b^, Siling Wang^a^*

*a School of Pharmacy, Shenyang Pharmaceutical University, 103 Wenhua Road, Shenyang, 110016, P. R. China.*

*b Beijing National Laboratory for Molecular Sciences, College of Chemistry and Molecular Engineering, Peking University, Beijing, 100871, P. R. China*

* Corresponding author.

Siling Wang Mail address: School of Pharmacy, Shenyang Pharmaceutical University, No.103 Wenhua Road, Shenyang 110016, P.R. China;

Email address: silingwang@syphu.edu.cn


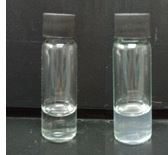


**Fig. S1** Macroscopic photos of β-CD solution before (left) and after (right) joining with Tween 60.


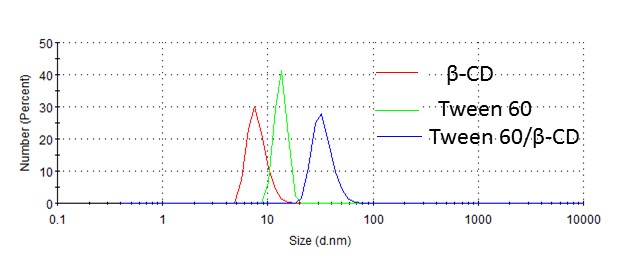


**Fig. S2** The particle size of Tween 60/β-CD, Tween 60 and β-CD measured by Zetasizer Nano.


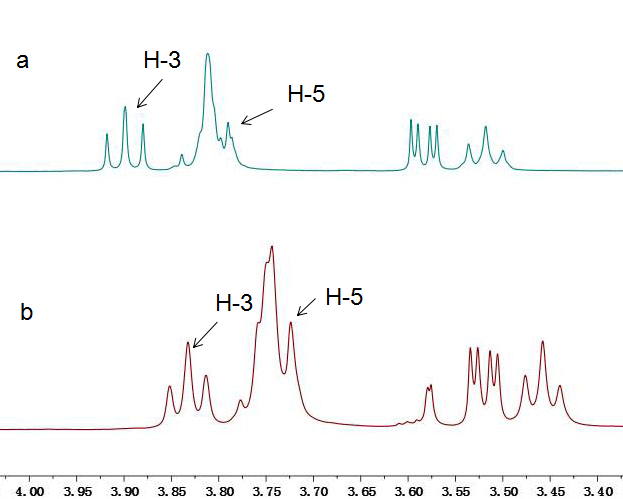


**Fig. S3** 1H-NMR spectra of β-CD in the absence (a) and presence (b) of Tween 60.


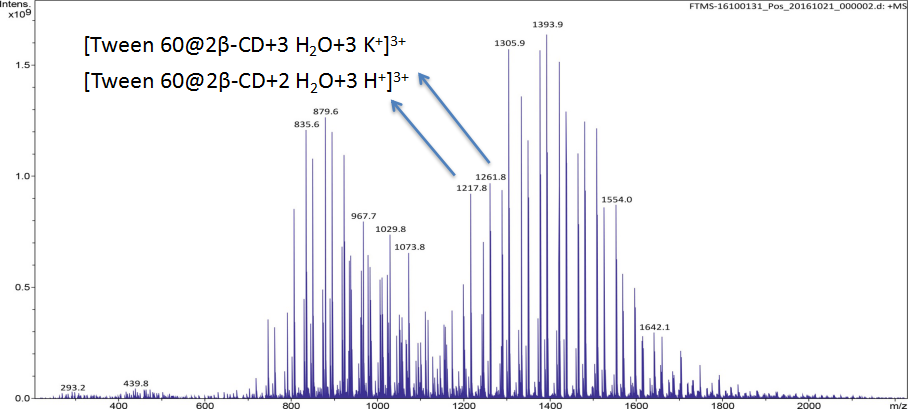


**Fig. S4** The ESI-MS results of Tween 60/β-CD complexes in positive mode.


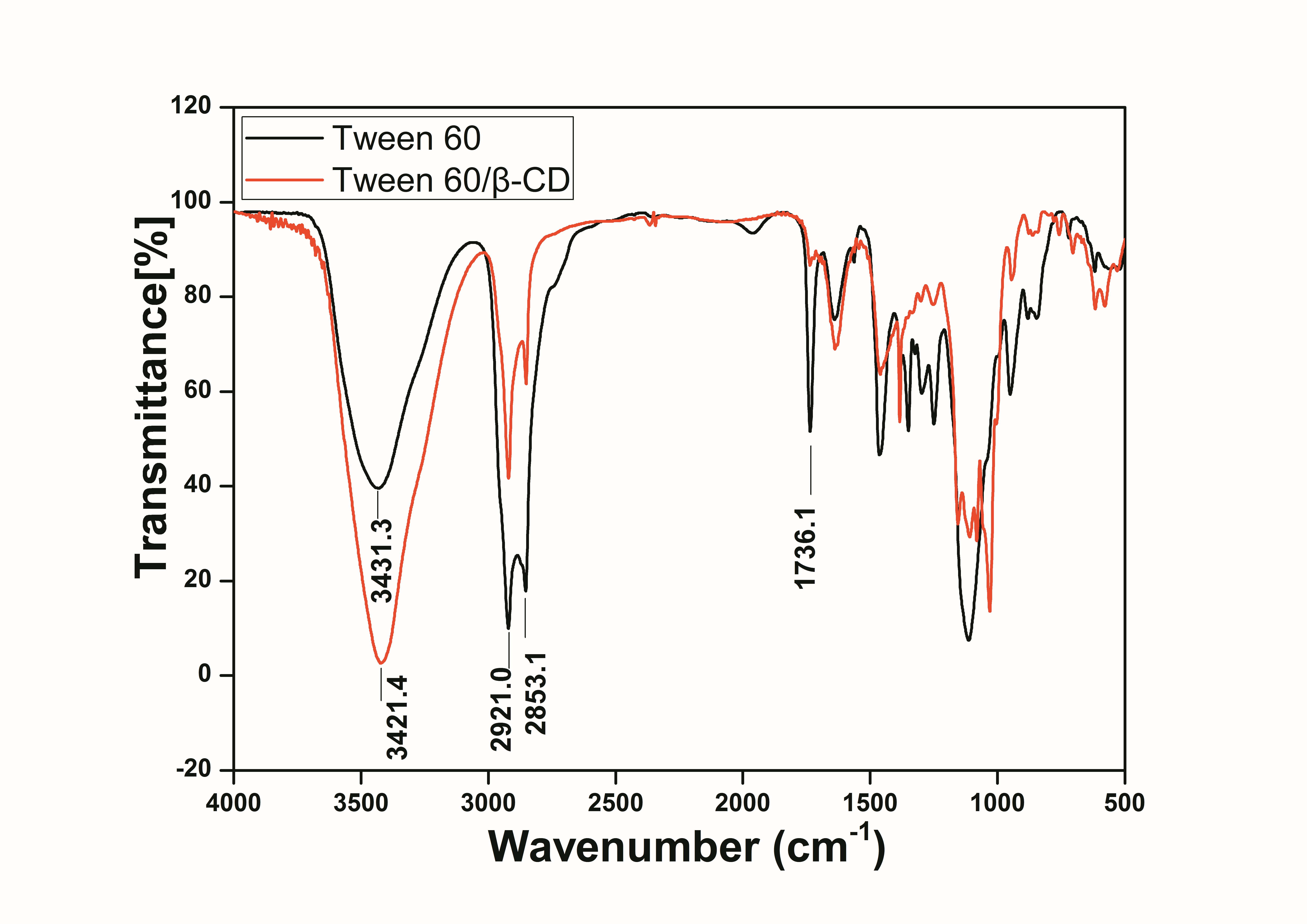


**Fig. S5** IR image of Tween 60/β-CD vesicles and Tween 60.


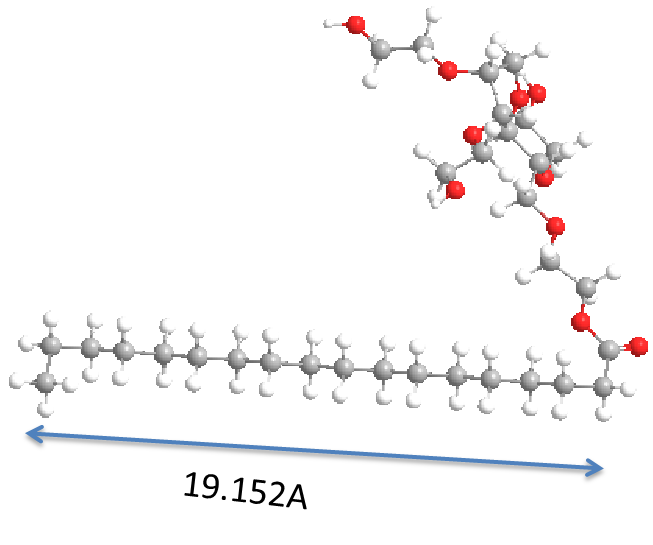


**Fig. S6** 3D simulation image of the molecular structures of Tween 60.

| Model | Equation | Equation pH 5.8 | R ^2^ | Equation pH 7.4 | R ^2^ |
| --- | --- | --- | --- | --- | --- |
| Zero order | R=kt+C | R=0.1187t+0.2305 | 0.8983 | R=0.0594t+0.234 | 0.8965 |
| First order | In(1-R)=kt+C | In(1-R)=-0.2681t-0.201 | 0.9798 | In(1-R)=-0.1018t-0.2504 | 0.9338 |
| Higuchi | R= kt**^1/2^** +C | R=0.3475t**^1/2^** +0.0293 | 0.9814 | R=0.2007t**^1/2^** +0.0888 | 0.9722 |
| Riger-Peppas | InR=kInt +C | InR=0.5039Int-0.9899 | 0.9879 | InR=0.398Int -1.2522 | 0.9899 |

**Table S1.** *In vitro* release kinetics parameters.


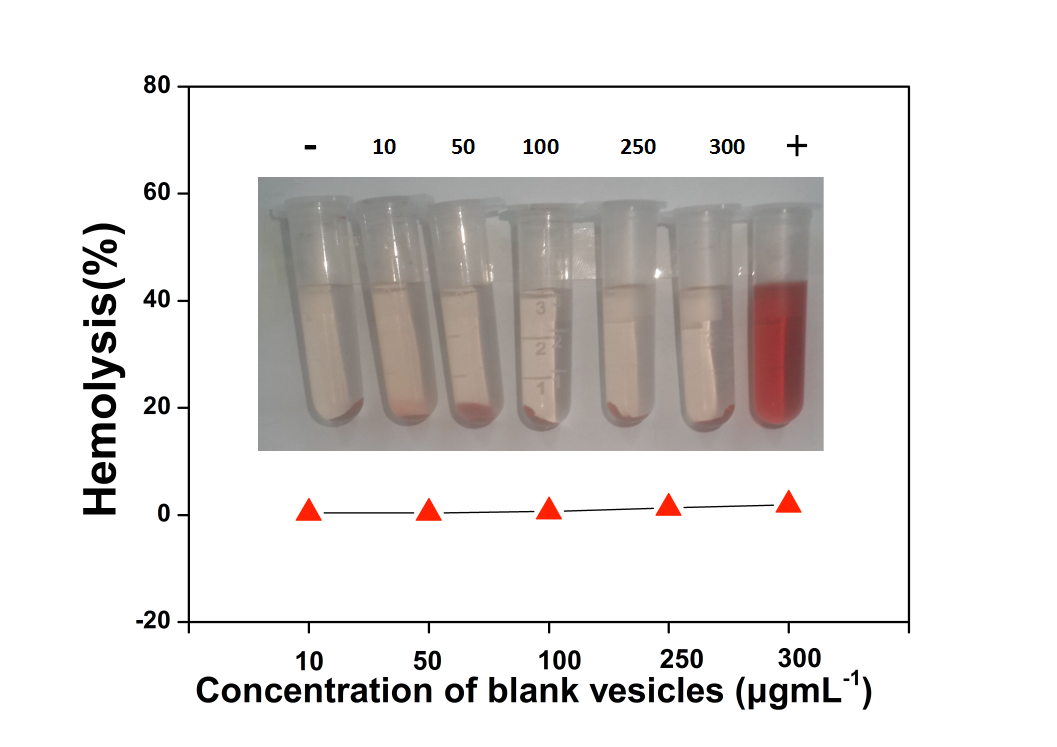


**Fig. S7** Hemolytic activity of Tween 60/β-CD vesicles at various concentrations. +: positive control. -: negative control.


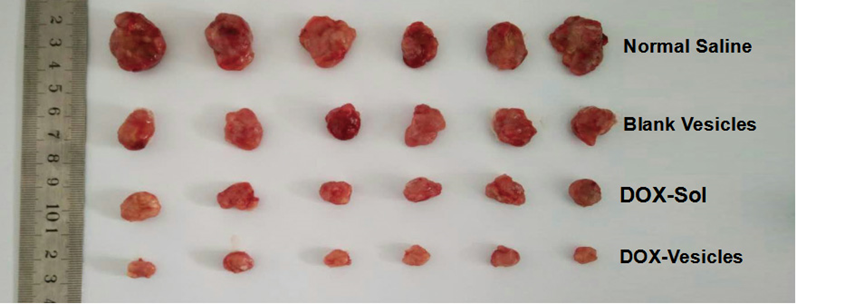


**Fig. S8** Representative images of excised tumors from the euthanized mices at 10th day.
